# Optical Coherence Tomography Assessment Before and After Vitamin Supplementation in a Patient With Vitamin A Deficiency: A Case Report and Literature Review

**DOI:** 10.1097/MD.0000000000002680

**Published:** 2016-02-12

**Authors:** Manuel Saenz-de-Viteri, Luis M. Sádaba

**Affiliations:** From the Department of Ophthalmology, Universidad de Navarra, Pamplona, Navarra, Spain.

## Abstract

Vitamin A is an essential fat-soluble vitamin important for the function of various body systems. In the eye, vitamin A is essential for the synthesis of visual pigments in photoreceptors. Vitamin A deficiency is a rare condition in the developed countries and might follow bariatric or intestinal bypass surgery.

We present the case of a 67-year-old male that complained of visual loss and nyctalopia. Patient had bariatric surgery 15 years before for weight loss. Low serum levels of vitamin A confirmed the diagnosis and patient started vitamin A supplementation. Visual fields, macular thickness, and ganglion cell layer thickness were recorded and monitored 1 month, 6 months, and 1 year after the beginning of therapy. Visual fields were significantly altered and central macular thickness and ganglion cell layer thickness were reduced, but the first 2 showed a significant recovery with vitamin supplementation therapy. By the 1st month of treatment patient referred a complete remission of visual symptoms. Further, we observed hyperreflective material accumulating beneath a partially disrupted ellipsoid band in the high definition optical coherence tomography that also improved progressively with vitamin repletion.

Newer and more sophisticated imaging systems have increased our knowledge of the mechanisms responsible for retinal diseases. To our knowledge, this is the first description of the effect of vitamin A deficiency and vitamin supplementation on macular thickness. This case also highlights the importance of considering bariatric bypass surgery as a cause of vitamin A deficiency in developed countries.

## INTRODUCTION

Vitamin A is an essential fat-soluble vitamin needed for the function of various body systems.^1,2^ This vitamin is required for immunity, gene transcription, and maintaining skin health.^1,3,4^ In the eye, vitamin A is essential for the normal retinal function where it is vital for the synthesis of visual pigments in both rods and cones during phototransduction.^5^ Further, vitamin A plays an important role in corneal and conjunctival epithelial cell RNA and glycoprotein synthesis.^1^ If left untreated, this nutritional deficiency could progress to cause permanent visual loss.^6,7^ Prolonged malnutrition, malabsorption, or abnormal metabolism can lead to vitamin A deficiency and visual symptoms.^7–9^

Vitamin A deficiency is a rare condition in the developed countries where malabsorption or abnormal metabolism are usually the cause.^2,10^ Several authors have reported cases of vitamin A deficiency as a result of bariatric or intestinal bypass surgery.^2,5,10,11^ A study of malabsortive bariatric surgery found an incidence of vitamin A deficiency of 69% by the 4th year after surgery.^12^

Here, we report a case of vitamin A deficiency in a patient with bariatric surgery 15 years prior to the beginning of ophthalmic symptoms. Visual fields, macular thickness, and Ganglion cell plus inner plexiform layer (GCIPL) thickness were monitored before and 1 month, 6 months, and 1 year after vitamin supplementation therapy.

## CASE PRESENTATION

**FIGURE 1:**
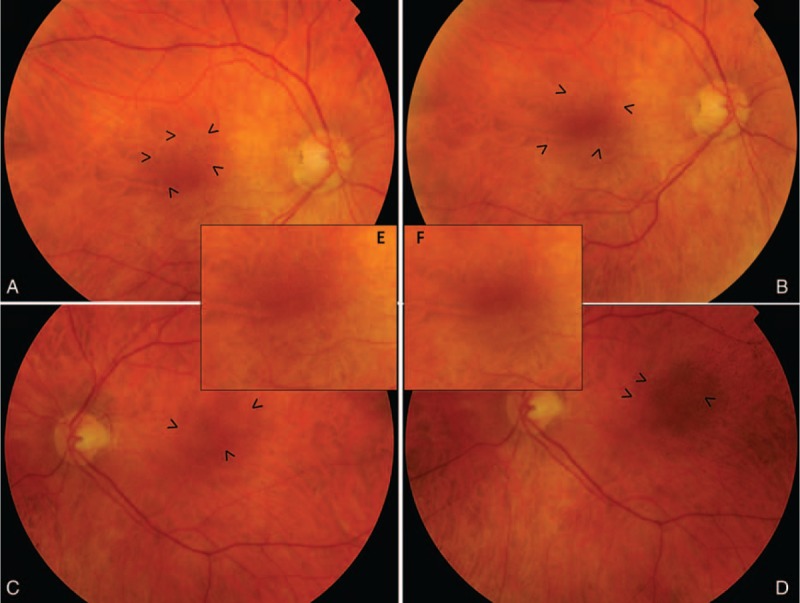
Retinography. Fundus appearance of the patient at the time of diagnosis (A: right eye and C: left eye). Punctate yellowish white spots in the macular region are seen in both eyes (arrow heads) and remained unchanged 1 y after vitamin supplementation therapy (B: right eye and D: left eye). Magnified macular region of the right eye (E) and left eye (F) can be observed.

**FIGURE 2:**
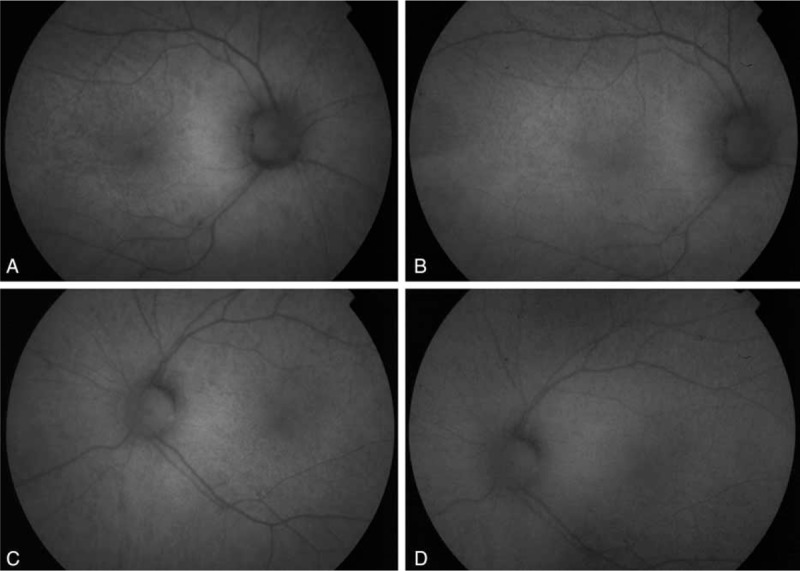
Fundus autoflorescence images. Fundus autoflourescence imaging showing a disuse mottled hypoautofluorescent pattern in both eyes before (A: right eye and C: left eye) and 1 y after the start of vitamin A treatment (B: right eye and D: left eye).

**FIGURE 3:**
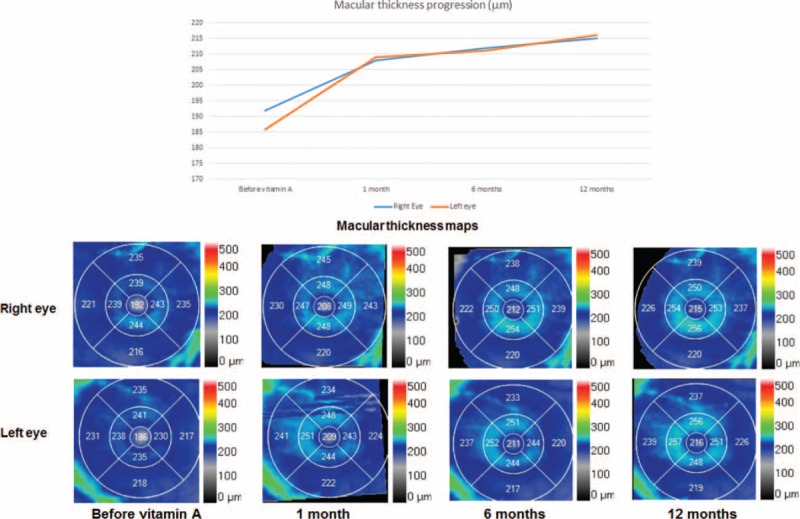
Macular thickness before and after vitamin supplementation. (A) Graph showing the evolution of macular thickness. (B) Macular thickness maps show a significantly reduced central thickness in both eyes at the time of diagnosis that progressively increased after the start of therapy.

**FIGURE 4:**
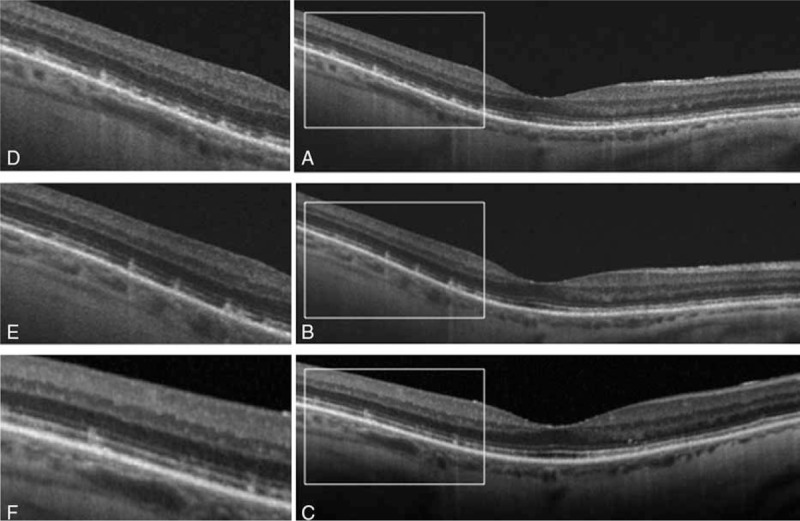
HD-OCT findings. The square highlights the hyperreflective material accumulating beneath a partially disrupted ellipsoid band at the time of diagnosis (A). OCT at 6 mo (B) and 1 y (C) show that, although some hyperreflective material remained visible beneath the ellipsoid band, the general appearance of the outer retinal layers improved progressively after the beginning of therapy. An enlarged image at each time point is shown (D–F). HD-OCT = high definition optical coherence tomography.

**FIGURE 5:**
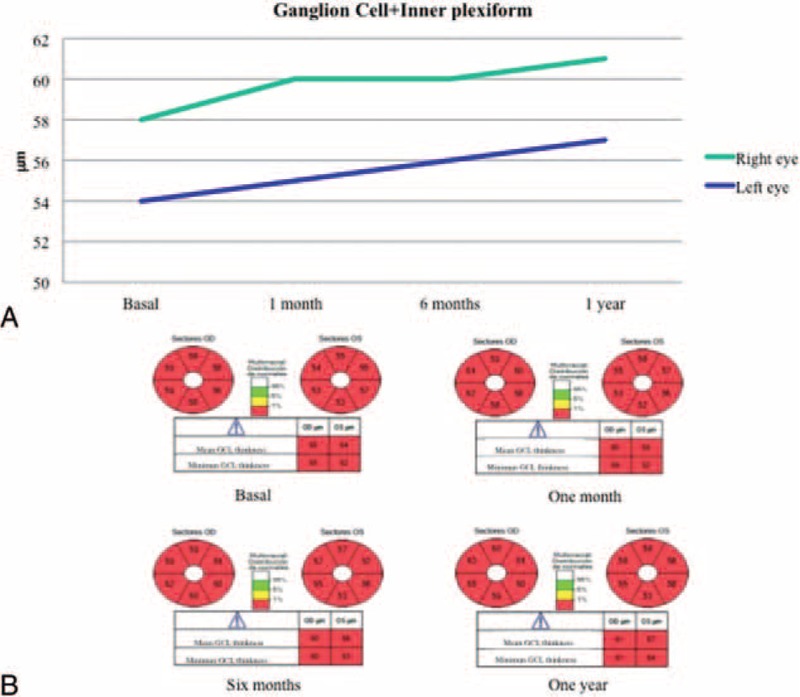
Ganglion cell plus inner plexiform layer thickness. (A) Graph showing the evolution of ganglion cell layer thickness. (B) Ganglion cell layer thickness was reduced in both eyes and did not vary significantly after vitamin supplementation.

**FIGURE 6:**
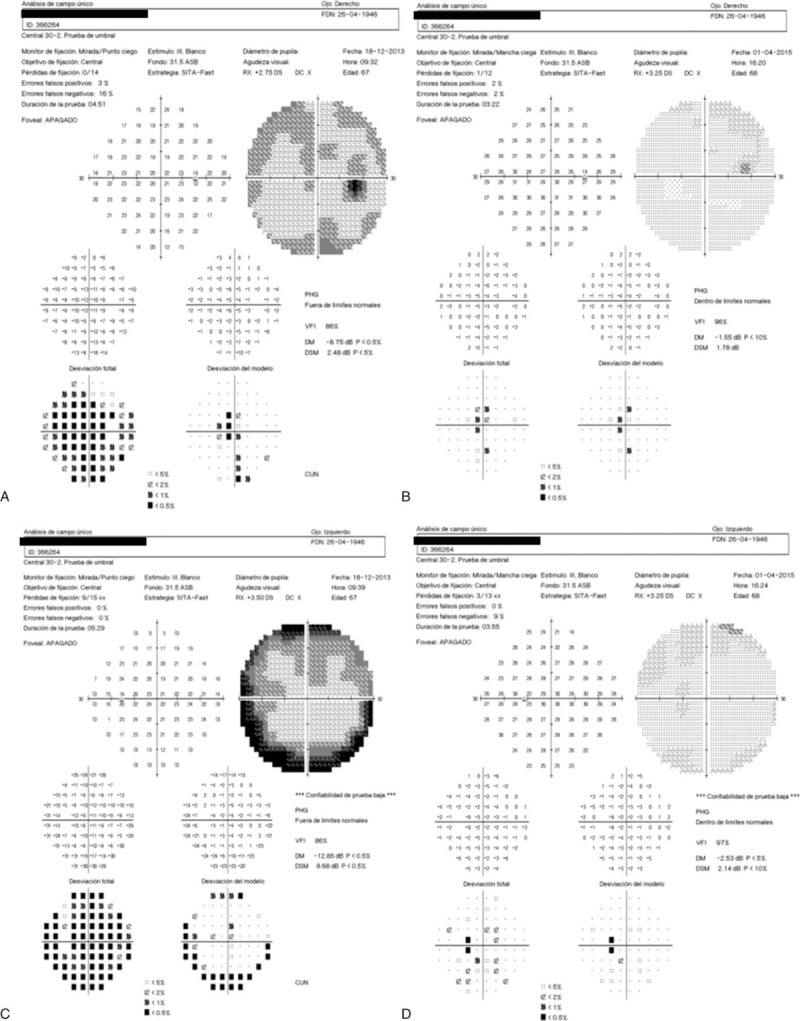
Visual fields before and after vitamin supplementation. Altered visual fields were detected at the time of the diagnosis (A: right eye and C: left eye), that significantly improved after vitamin A supplementation (B: right eye and D: left eye).

A 67-year-old male presented to our hospital with a 5-month history of vision loss in both eyes that was significantly worse at night. Medical history was unremarkable except for a bariatric surgery 15 years before for weight loss. Visual acuity was 1.0 in the right eye and 0.9 in the left eye and anterior segment was unremarkable. Fundoscopy revealed multiple punctate yellowish white spots in the macular region (Figure 1) and fundus autoflourescence (FAF) imaging showed a mottled hypoautofluorescent pattern (Figure 2). Spectral domain optical coherence tomography (SD-OCT) images were obtained and macular thickness was significantly reduced in both eyes (Figure 3). Further, we observed hyperreflective material accumulating beneath a partially disrupted ellipsoid band in the high definition optical coherence tomography (HD-OCT) (Figure 4). GCIPL was reduced in both eyes (mean GCIPL 58 μm in the right eye and 54 μm in the left) (Figure 5). Visual fields were also altered bilaterally (Figure 6). Ocular findings together with nyctalopia and the history of previous bariatric surgery led to the suspicion of vitamin A deficiency. Serum levels of vitamin A were 0.08 mg/L (normal range 0.2–0.5 mg/L) confirming the diagnosis. Levels of other fat-soluble vitamins were also significantly reduced: Vitamin D 25-OH 9.58 ng/mL (20–75 ng/mL), vitamin E 2.70 μg/mL (5–20 μg/mL). Patient began treatment with intramuscular vitamin A followed by oral supplementation with 50,000 IU of retinol palmitate daily. Vitamins D and E deficiencies were also treated with calcifediol 266 μg every 48 h and tocopherol acetate 400 mg every 24�hours. Patient was monitored 1 month, 6 months, and 1 year after the beginning of therapy. A complete remission of the visual symptoms was achieved by the 1st month with vitamin A levels within the normal range (0.3 mg/L). Visual fields also showed a significant recovery and central macular thickness had increased in both eyes. Both parameters continued to improve in the subsequent visits (Figures 2 and 5). Fundoscopic appearance and FAF remained unchanged (Figures 1 and 2). Similarly, GCIPL did not vary significantly (Figure 5). Although some hyperreflective material remained visible beneath the ellipsoid band, the general appearance of the outer retinal layers also seemed to improve progressively after the beginning of therapy (Figure 4). Patient continues long-term treatment with 50,000 IU of retinol palmitate daily, calcifediol and tocopherol at the doses described above. Informed consent to publish this report was obtained from the patient.

## DISCUSSION

Although vitamin A deficiency has largely disappeared in relatively wealthy populations, it remains a prevalent problem in the developing world.^6^ An estimated 10 million preschool-age children and pregnant women develop potentially blinding xerophthalmia each year and vitamin A deficiency is estimated to be responsible for about half million cases of irreversible blindness each year.^6,7^ On the contrary, the higher prevalence of obesity in developed countries has increased the rate of iatrogenically induced malabsorption syndromes and vitamin A deficiency in patients who undergo bariatric surgery.^8,11^

Similar to our case, vitamin A deficiency might present even decades after bariatric surgery and despite oral vitamin supplementation.^10^ Nyctalopia, the main complain of our patient is 1 of the earliest and most common symptom of vitamin A deficiency.^2,5,9,10^ Night blindness usually resolves within the 1st weeks after vitamin supplementation therapy.^10^

In this report, we describe some known changes that are commonly found in patients with vitamin A deficiency, but we also show some new structural abnormalities that can now be assessed with the use of better imaging technologies like OCT.

Our patient also presented with altered visual fields that improved soon after vitamin supplementation was started. Other authors have described superior and inferior arcuate defects or concentric narrowing of the visual field in patients with vitamin A deficiency.^5,10^ However, visual fields’ defects are not a constant finding in this condition.^5,10^

There is limited information on the fundus findings and their response to vitamin A therapy.^13^ Multiple retinal yellowish white spots like the ones present in our patient have been described in other cases with vitamin A deficiency.^5,9,10,13^ Some authors have reported that similar lesions have resolved 2 or 3 months after vitamin supplementation.^13^ In our case, this spots were still visible 1 year after the beginning of treatment. Aleman et al^9^ described that these lesions colocalized with hypoautofluorescent round areas on FAF. Further, they analyzed these spots by SD-OCT and found hyperreflective images that accumulated beneath the ellipsoid band replacing the photoreceptor outer segment signal.^9^ They suggest that the white lesions correspond to a disruption and accumulation of shed photoreceptor outer segment above the retinal pigmented epithelium and that localized loss of lipofuscin could explain the hypoautofluorescent spots observed in these patients.^9^ In our case, the yellowish punctate spots were limited to the macular region but the areas of hypoautoflourescence were more numerous and spread throughout the posterior pole and mid periphery. Our OCT findings were similar to Alemans, as we also found hyperreflective material accumulating beneath a partially disrupted ellipsoid band. However, to our knowledge we are the first to describe a decrease in macular thickness that partially recovered after vitamin supplementation.

Ganglion cell layer abnormalities like the reduction found in our case have not been described before in patients with vitamin A deficiency. Although the effect of vitamin A deficiency on the inner layers of the retina remains unclear, a recent study in rats analyzed the effect of vitamin A deficiency in the oscillatory potentials of the electroretinogram (reflects the function of amacrine and ganaglion cells) and found a reduction of these potentials associated with vitamin A deprivation.^14^

To our knowledge, this is the first report macular thickness in a patient with vitamin A deficiency and it response to supplementation therapy. Further, our findings are similar to a previous OCT reports of structural damage in the outer layers of the retina. We also found a decrease in the ganglion cell layer thickness that was not significantly modified with treatment. Newer and more sophisticated imaging systems have increased our knowledge of the mechanisms responsible for retinal diseases. More studies are needed to confirm these findings in more patients suffering from this potentially blinding disease.
